# Primary Cutaneous Apocrine Carcinoma of the Thigh: A Rare Case Report

**DOI:** 10.7759/cureus.57859

**Published:** 2024-04-08

**Authors:** Meriem Bouabid, Ahmed BenSghier, Mohamed Moukhlissi, Soufiane Berhili, Loubna Mezouar

**Affiliations:** 1 Department of Radiation Oncology, Centre Hospitalier Universitaire Mohammed VI, Oujda, MAR

**Keywords:** chemotherapy, radiotherapy, surgical resection, adnexal carcinoma, primary apocrine

## Abstract

Adnexal carcinomas are rare cutaneous malignancies arising from the eccrine and apocrine sweat glands, follicles and sebaceous glands. They occur mainly in elderly people. We report the case of a patient treated for locally advanced apocrine adnexal carcinoma of the thigh, with a review of the literature.

The patient was 69 years old, he complained of pruritus on the anterior region of the left thigh four months ago with the appearance of a homolateral inguinal mass without any other associated signs. A left inguinal adenectomy was performed. After almost 15 days, the left inguinal adenopathy reappeared associated with diffuse erythematous nodules on the anterior region of the thigh. The pathological study suggested an adnexal carcinoma of the apocrine type. Surgical treatment was not feasible, therefore primary exclusive radiotherapy was administered at a total dose of 70 Gy in 35 fractions of 2 Gy each. Radiotherapy went well with some adverse events. One-month post-radiation assessment showed clinical and radiological progression.

## Introduction

Adnexal carcinomas are rare cutaneous malignancies arising from the epithelial appendages of the skin. They are divided into four groups: carcinomas arising from eccrine glands, apocrine glands, follicular structures and sebaceous glands [1]. Apocrine-type adnexal carcinomas occur at an average age of 66 years. There is no difference in frequency between the sexes [2].

Apocrine carcinomas are frequently localized in the axillary region which contains the greatest concentration of apocrine glands or in the anogenital region. They can also be found in the head and neck, the trunk or less frequently in the limbs [2]. 

The etiology remains unknown, but certain risk factors that may favor their carcinogenesis are ionizing radiation, exposure to ultraviolet rays, immunosuppression and antecedents of trauma [2,3].

We report the case of a male patient treated at the Hassan II Oncology Center of Oujda for an apocrine adnexal carcinoma of the thigh, with a review of the literature to assess the epidemiological, clinical, therapeutic and prognostic features of this pathology.

## Case presentation

The patient was 69 years old and had undergone surgery two years ago for basal cell carcinoma of the left leg with good control. Four months ago, he complained of pruritus on the anterior region of the left thigh with the appearance of a homolateral inguinal mass without any other associated signs.

A left inguinal adenectomy was performed with an anathomopathological study returning in favor of a lymph node metastasis of a poorly differentiated carcinomatous process; the immunohistochemical study (anti-CK 7 antibody positive, anti-CK 20 antibody positive, anti-PS 100 antibody positive, anti-CK19 antibody positive, TTF 1 antibody negative and anti-CDx2 antibody negative) was inconclusive.

**Figure 1:**
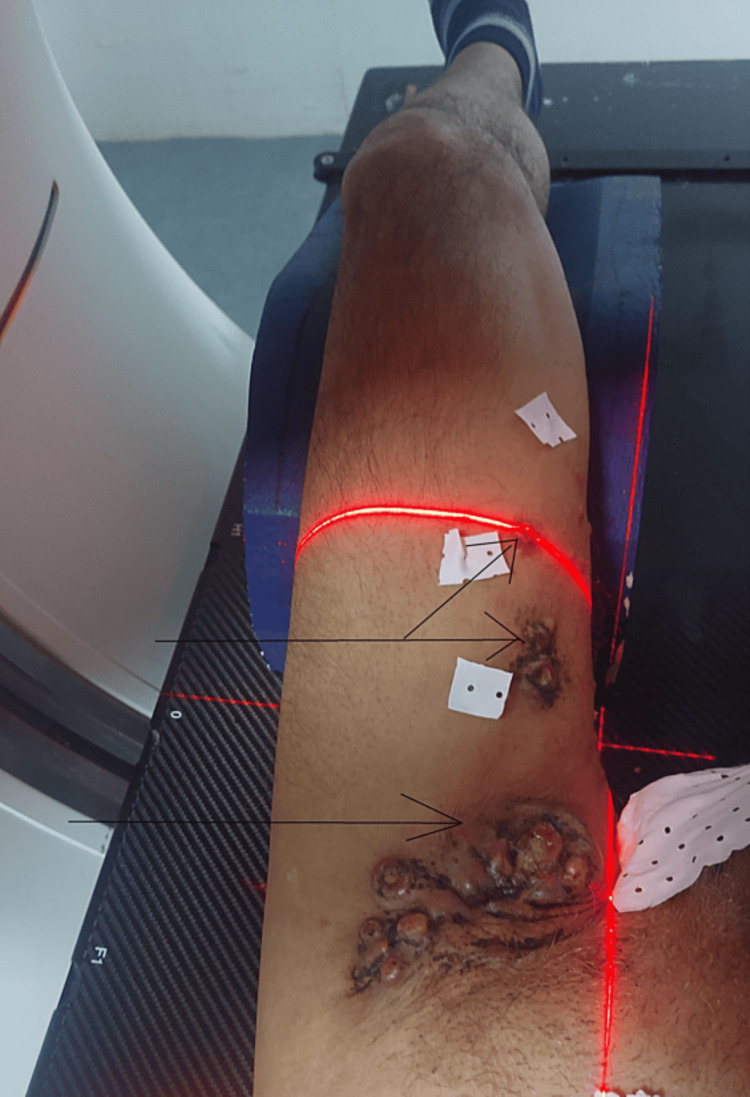
Picture showing diffuse erythematous nodules on the anterior region of the thigh with a left inguinal adenopathy (black arrows)

**Figure 2:**
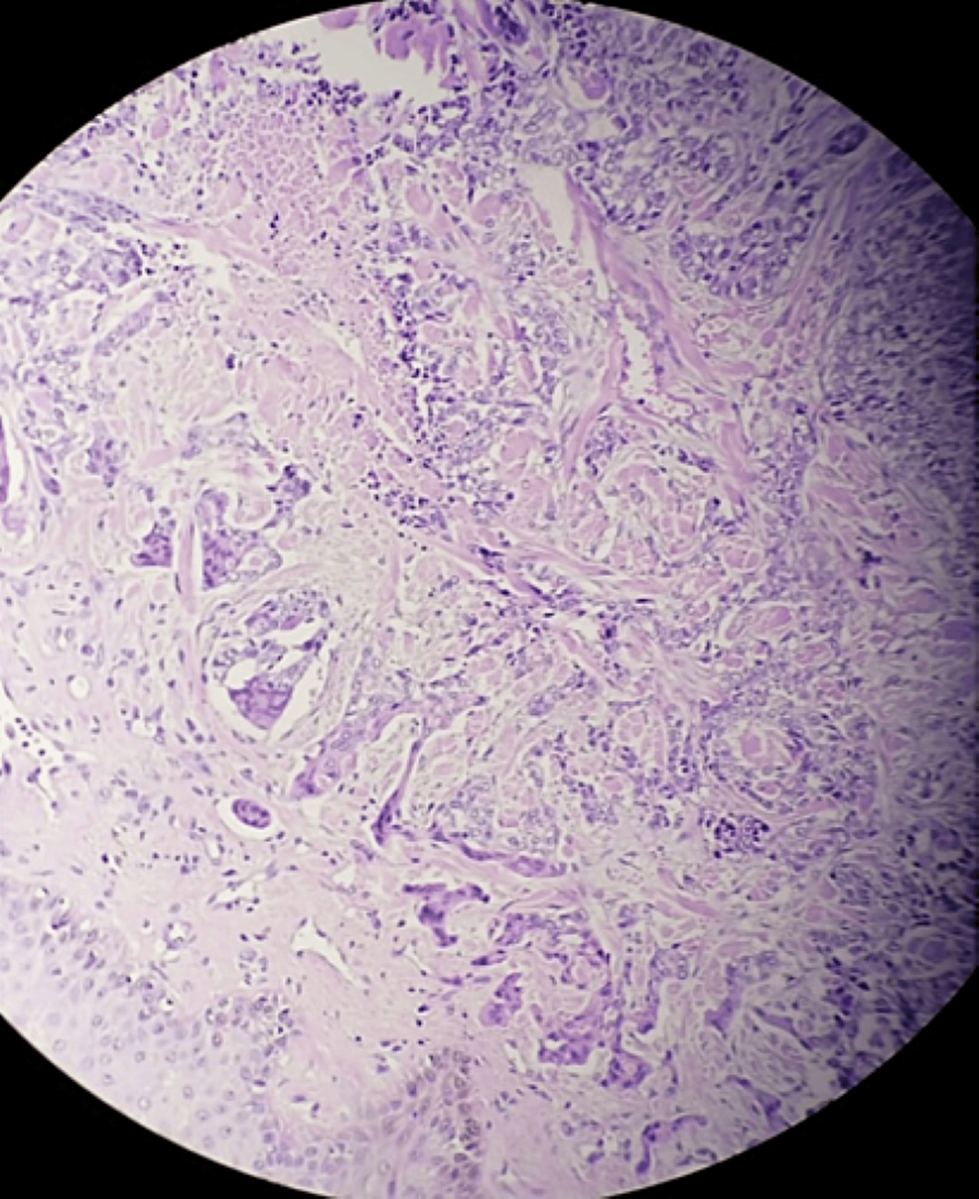
Photomicrograph of the lesion showing adnexal carcinoma of the apocrine type.

After almost 15 days, the left inguinal adenopathy reappeared associated with diffuse erythematous nodules on the anterior region of the thigh (Figure 1). The patient was therefore reoperated and the pathological study found an adnexal carcinoma of the apocrine type (Figure 2).

**Figure 3:**
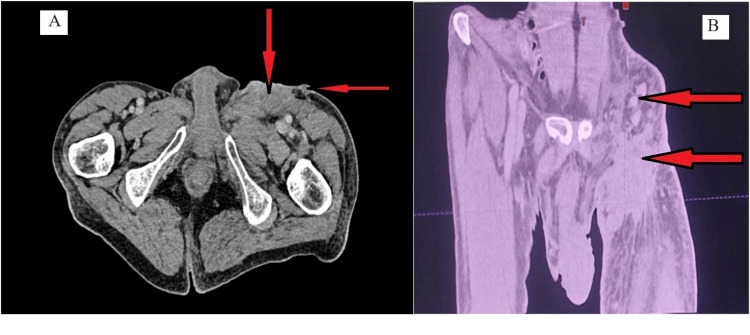
Axial (A) and coronal (B) slice of injected scan CT of the thigh, showing subcutaneous tumor mass on the anterior region of the thigh extending towards the left inguinal region measuring 66 x 30 mm, with the presence of nodular skin lesions (red arrows)

**Figure 4:**
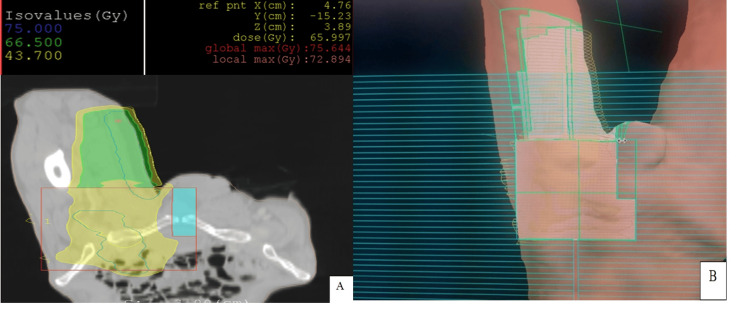
(A) and (B), showing the treatment planning of the subcutaneous tumor mass on the anterior region of the thigh extending towards the left inguinal region with the presence of left inguinal adenopathy and a few nodular skin treated by a bolus (shown in green).

A CT scan of the thigh (Figure 3) was performed, revealing a subcutaneous tumor mass on the anterior region of the thigh extending towards the left inguinal region measuring 66 x 30 mm, with the presence of left inguinal adenopathy and a few nodular skin lesions. No distant secondary lesions were found on the thoracic-abdominal-pelvic CT scan. Therefore the diagnosis of locally advanced primary apocrine adnexal cutaneous carcinoma was made in our patient. Surgical treatment was not feasible, so primary exclusive radiotherapy was realized at a total dose of 70 Gy in 35 fractions of 2 Gy each (Figure 4).

Radiotherapy was successful following the effective management of skin nodule infection with antibiotics, and the grade II radiodermatitis improved with medical treatment. One-month post-radiation assessment showed a clinical and radiological progression with the appearance of distant metastases.

## Discussion

Adnexal carcinomas are rare malignant skin tumors; they represent just 1% of all skin tumors. They arise from the pilosebaceous epithelial appendages and from the eccrine and apocrine sweat glands. They are characterized by locoregional and distant extension [1,2].

Apocrine adnexal carcinoma occurs at an average age of 66 years [4]. There is no difference in frequency between the sexes [3,5,6]. Clinically, they appear as asymptomatic nodules or plaques with a slowly progressive evolution; they may reach large diameters and ulcerate [1,7]. Some patients present with involved regional lymph nodes at diagnosis or even metastases to the lungs, liver, bone or brain [8]. 

Biopsy and pathological study are necessary to confirm the diagnosis. The histological image is highly characteristic. The epithelial component of these carcinomas is organized into tubular and papillary structures. The cells are generally eosinophilic, and the diagnosis of an apocrine tumor is made when images of decapitation secretion are observed. Deep invasive architecture, asymmetry, high mitotic activity and nuclear pleomorphism make the diagnostic of malignancy [1,7].

Immunohistochemistry is essential in cases of diagnostic doubt with other types of adnexal carcinoma or with metastatic apocrine carcinoma of the breast. Therefore, in the case of primary apocrine carcinoma, we found that the cells are positive for cytokeratins CK5/6 and CK7, P63, podoplanin, GCDFP-15 and sometimes are also positive for ostrogenic or progesterone hormone receptors [6,7,9]. However, they are negative for mammaglobin and HER2 [10].

The therapeutic approach is based on surgical resection of the tumor with negative margins; although the margin's extent is not clarified; some authors suggest that the margin should be between 1 and 2 cm more or less associated with lymph node curage [6,11,12]. 

The adjuvant radiotherapy has a great benefit in the therapeutic strategy, in cases of risk factors for recurrence, namely the presence of perineural invasion, lymph node invasion, positive surgical margins or recurrent tumor. And it’s generally delivered at a total dose of 60 Gy in 30 fractions of 2 Gy or a total dose of 50 Gy in 20 fractions of 2.5 Gy, in order to ensure a good locoregional control [13,14]. 

In the case of refusal surgery or extensive non-resectable tumors, as in our patient's case, exclusive radiotherapy may be indicated at a total dose of 66-70 Gy in fractions of 2 Gy. Neoadjuvant radiotherapy has been described in few papers with a low level of proof [14]. 

Volumes and margin for radiotherapy must consider the infiltrative nature of the tumor. For lymph nodes, areas invaded should be irradiated; and only the ipsilateral side can be irradiated for a prophylactic purpose, especially when lateralised tumour, like it is currently done for skin squamous cell carcinoma [14,15]. 

Chemotherapy has little place in the adjuvant treatment of apocrine adnexal carcinomas; more studies are needed to demonstrate its benefit [13]. However, it may be indicated in very advanced and metastatic cases, or in cases of recurrence [11]. 

The prognosis of primary apocrine carcinoma depends principally on lymph node involvement. Ten-year disease-free survival rates are reported to be 56% in negative lymph node cases. However, this percentage decreases to 9% if there is lymph node metastases [16,11]. 

## Conclusions

Apocrine adnexal carcinoma is a very rare tumor; only a few cases have been reported in the literature. The treatment of choice is local excision with clear margins, with or without lymph node dissection. Radiotherapy allows significant improvement of local control in the adjuvant setting and constitutes the only curative treatment in non-resectable cases.
